# Unveiling GDF-15: a new frontier in combating diabetic osteoporosis

**DOI:** 10.3389/fmed.2026.1819289

**Published:** 2026-04-15

**Authors:** Nan Wang, Yingyi Gao, Ning Bai

**Affiliations:** 1 Department of Medicine, Wuxi Medicine College, Jiangnan University, Wuxi, Jiangsu, China; 2 Department of Endocrinology, Affiliated Hospital of Jiangnan University, Wuxi, Jiangsu, China

**Keywords:** bone metabolism, diabetic osteoporosis, growth differentiation factor-15, osteoblast, osteoclast

## Abstract

Diabetic osteoporosis (DOP) is a diabetes mellitus (DM) complication, defined by diminished bone density and a significantly increased fracture risk due to disorders in bone metabolism, making it a key clinical concern. Growth differentiation factor-15 (GDF-15), a transforming growth factor-β superfamily member, has broad target specificity and exerts diverse biological effects through multiple signaling pathways, drawing attention to its role in metabolic diseases. GDF-15 has been implicated in the regulation of osteoblast- and osteoclast-related processes. It is abnormally expressed in DOP patients and may be correlated with disease progression, potentially mediated by insulin resistance, hyperglycemic toxicity, ferroptosis, and inflammatory responses. Although some biological functions of GDF-15 have been elucidated, its specific pathways in this disease and clinical application value warrant further studies. This review summarizes recent advances in basic and clinical research, elucidates the pathophysiological mechanisms behind GDF-15 in DOP, and provides theoretical support and research directions for GDF-15-based targeted therapeutic strategies.

## Graphical abstract

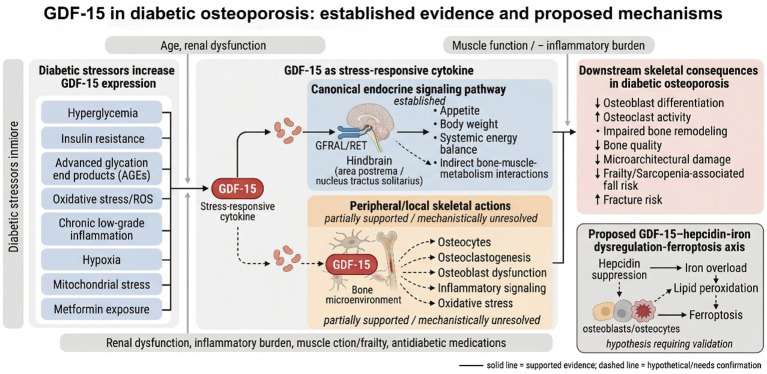

## 1 Introduction

Diabetic osteoporosis (DOP) is a severe complication of diabetes mellitus (DM), characterized by bone loss and structural disruption, increasing fracture risk, and affecting the quality of life. Its complex pathogenesis involves hyperglycemia, insulin resistance (IR), inflammation, osteoclast dysfunction, and microvascular issues (1, 2). Notably, fractures can occur even with normal or high bone density, indicating a unique osteoporosis pattern in DM (2). Type 1 diabetes mellitus (T1DM) is more commonly characterized by absolute insulin deficiency, insufficient bone mass acquisition, and impaired bone formation, whereas type 2 diabetes mellitus (T2DM) is often associated with relatively preserved bone density but reduced bone quality, increased deposition of advanced glycation end-products (AGEs), and an elevated risk of falls and frailty-related fractures (3, 4). Chronic inflammation and metabolic issues, driven by immune abnormalities and high inflammatory cytokines, lead to increased bone resorption and reduced formation, thereby disrupting bone metabolism (5). Moreover, DM-associated osteoporosis is characterized by the abnormal proliferation of bone marrow adipose tissue, which significantly affects the bone microenvironment and bone cell function through various cytokines and metabolic regulators’ secretion, thereby exacerbating bone loss (6). Recent studies have identified mechanisms, including the Wnt signaling pathway, iron metabolism abnormalities, and ferroptosis, as critical molecular underpinnings of diabetic bone disease (7, 8).

Additionally, gut microbiota alterations in DM patients are associated with increased intestinal permeability, systemic inflammation, and IR, all of which may contribute to abnormal bone metabolism (2). In the liver-bone axis, the hepatic factor lecithin-cholesterol acyltransferase is involved in the reverse transport of cholesterol from bones to the liver, with its deficiency potentially exacerbating bone loss (9). The “gut-bone” and “liver-bone” axes play complex roles in regulating bone metabolism through distinct pathways.

Growth differentiation factor-15 (GDF-15), a member of the transforming growth factor-β (TGF-β) superfamily, is also known as macrophage inhibitory cytokine-1, nonsteroidal anti-inflammatory drug-activated gene-1, placental bone morphogenetic protein, and prostate-derived factor. The amino acid sequence of GDF-15 shares homology with the *osteomorphin* gene. The gene encoding GDF-15 resides on chromosome 19p13.1 and comprises two exons (309 and 891 bp) separated by a single intron (1820 bp) (10). GDF-15 is synthesized as a 308-amino acid precursor polypeptide containing a 29-amino acid signal peptide, a 167-amino acid propeptide, and a 112-amino acid mature domain, with an N-linked glycosylation site at position 70 (11). Under physiological conditions, GDF-15 is constitutively expressed at variable levels in epithelial cells of the placenta, prostate, mammary gland, kidney, liver, lung, colon, pancreas, and gastrointestinal tract (12, 13). GDF-15 influences a broad spectrum of biological pathways and contributes to aging, metabolism, and inflammation-related processes through various signaling mechanisms. The primary receptor for GDF-15 is the glial cell line-derived neurotrophic factor receptor alpha-like (GFRAL) protein, which complexes with the rearranged during transfection (RET) co-receptor to regulate appetite and energy homeostasis within the central nervous system (14–16). GDF-15 primarily activates intracellular signaling pathways through Drosophila mothers against decapentaplegic (SMAD)-dependent and -independent mechanisms. Upon binding to the GFRAL/RET complex, GDF-15 modulates classical TGF-β signaling, activates SMAD2/3, and regulates processes, including cell proliferation, apoptosis, and inflammatory responses. Additionally, GDF-15 affects non-SMAD signaling pathways, including phosphoinositide 3-kinase/protein kinase B/mitogen-activated protein kinase/extracellular signal-regulated kinase (ERK), and signal transduction and transcription-activating protein 3, thereby contributing to cellular metabolic regulation, immune responses, and cell survival (17, 18). Under specific tissue and pathological conditions, GDF-15 exerts diverse biological effects through the aforementioned signaling pathways. In the cardiovascular system and kidneys, for example, GDF-15 exerts protective effects by modulating these pathways, thereby reducing tissue injury and inflammation (19, 20). Regarding bone metabolism, existing studies suggest that the regulatory role of GDF-15 in osteogenic differentiation remains somewhat controversial. An *in vitro* study using MC3T3-E1 pre-osteoblasts found that GDF-15 can negatively regulate osteogenic differentiation by inducing Protein Phosphatase 2A Catalytic Subunit Alpha (PP2A Cα) to upregulate Nucleoredoxin-like 1 (NXNL1), thereby inhibiting Bone Morphogenetic Protein 2 (BMP2)-induced Smad1/5/9 phosphorylation and alkaline phosphatase (ALP) activity (21). In contrast, another study on ankylosing spondylitis demonstrated that GDF-15 promotes osteogenic differentiation by activating the Wnt/β-catenin signaling pathway (22). Additionally, GDF-15 is vital for regulating energy balance by inducing appetite suppression and weight loss, while exerting anti-inflammatory, antioxidant, and anti-fibrotic effects in peripheral tissues. The complexity and diversity of the GDF-15 signaling pathway make it a key regulator of various metabolic diseases and their associated complications. (23, 24). It should be noted that the most clearly defined classical receptor pathway to date is the GDF-15/GFRAL/RET axis, and GFRAL expression is primarily restricted to the area postrema and nucleus tractus solitarius in the brainstem. Therefore, the direct mechanism of action of GDF-15 in peripheral bone tissue has not yet been fully elucidated; currently, it should be cautiously described as a potential local paracrine or alternative signaling effect, rather than a fully established classical receptor-mediated effect (25, 26).

## 2 GDF-15 expression regulation in diabetic conditions

From a broad perspective of metabolic biology, GDF-15 functions more as a stress-responsive cytokine, which is regulated by mitochondrial stress, oxidative stress, endoplasmic reticulum stress, and integrated stress responses. Therefore, in the context of diabetes-related bone diseases, the upregulation of GDF-15 may reflect either a compensatory adaptation or persistent pathological stress; its biological significance is clearly context-dependent (27, 28). Serum GDF-15 concentrations are markedly elevated in individuals with DM and correlate strongly with impaired glucose metabolism, chronic inflammation, and the progression of diabetic complications (29, 30). A cross-sectional study in a Chinese cohort demonstrated a positive association between circulating GDF-15 levels and both DM prevalence and IR; notably, the GDF-15-to-adiponectin ratio exhibited high predictive accuracy for type 2 DM (T2DM) (29). Furthermore, a systematic review and meta-analysis indicated a nonlinear positive relationship between circulating GDF-15 concentrations and the prevalence of DM, which plateaus following surpassing a certain threshold (31). In diabetic conditions, pro-inflammatory factors, oxidative stress, and hyperglycemic environments are the primary drivers of increased GDF-15 expression. Hyperglycemia triggers cellular stress responses, leading to GDF-15 transcription, thereby establishing it as a diabetic stress biomarker (14, 20). Moreover, plasma GDF-15 levels positively correlate with DM-associated inflammatory markers: interleukin (IL)-6 and tumor necrosis factor-alpha (TNF-α) (30, 32). Research suggests that GDF-15 may protect against gestational DM by mitigating fetal overgrowth, although it is also associated with adverse perinatal outcomes (14, 32). Genetic factors participate in variations in GDF-15 expression levels among patients with DM, with specific GDF-15 gene polymorphisms significantly associated with an elevated T2DM risk and altered serum GDF-15 levels, indicating genetic regulation of GDF-15 expression (20). In addition, when interpreting elevated circulating GDF-15 levels in patients with diabetes, the effects of antidiabetic medications—particularly metformin—must also be considered. Previous studies have shown that metformin can significantly increase GDF-15 levels and contribute to weight management and metabolic regulation by enhancing GDF-15 secretion; therefore, drug exposure must be considered an important confounding factor in the clinical interpretation of GDF-15 levels in the diabetic population (22, 33, 34).

## 3 Mechanism of GDF-15 in regulating bone metabolism

### 3.1 GDF-15 effects on osteoblast function

GDF-15 is a vital regulator in bone metabolism, significantly affecting osteoblast proliferation, differentiation, and mineralization. Some studies suggest that GDF-15 may participate in and regulate bone formation through endocrine and paracrine mechanisms. Notably, elevated GDF-15 levels are commonly observed in those with osteoporosis, and this increase is inversely associated with bone density and quality. Research involving elderly female patients with osteoporosis revealed that serum GDF-15 concentrations were markedly higher in those with fractures compared to non-osteoporotic individuals. Additionally, GDF-15 mRNA expression was positively correlated with TNF-α and IL-1β, indicating that GDF-15 may indirectly inhibit osteoblast function by inflammatory pathways, thereby reducing bone formation (35). Moreover, increased GDF-15 levels are related to decreased muscle mass, which could further influence skeletal mechanical loading and osteoblast activity, establishing a negative feedback loop in bone-muscle interactions (36). It is worth noting that recent prospective studies have shown that elevated GDF-15 levels are associated with an increased risk of hip fractures in older adults. However, this association may be partially mediated by decreased muscle strength and weight loss, rather than directly through changes in bone density. This suggests that, in clinical practice, GDF-15 may reflect risks associated with the bone-muscle-frailty axis rather than acting solely as a bone-specific pathogenic factor (37).

### 3.2 Regulatory impacts of GDF-15 on osteoclasts

GDF-15 exerts a significant influence on osteoblasts and regulates osteoclast differentiation and activity, thereby affecting bone resorption. Research suggests that GDF-15 facilitates osteoclast differentiation by modulating osteoclast precursor cell maturation, thereby enhancing bone resorption capacity. In a hypoxic bone microenvironment, osteocyte-derived GDF-15 promotes osteoclast differentiation, suggesting its involvement in local bone resorption processes; however, this conclusion is currently based primarily on specific experimental models (35, 38). At the molecular level, this process specifically involves the regulation of receptor activator of nuclear factor κB ligand (RANKL)/osteoprotegerin (OPG) signaling. RANKL functions as a critical stimulatory factor for osteoclast differentiation, whereas OPG serves as its natural antagonist. The balance between these two molecules dictates the extent of bone resorption. GDF-15 promotes RANKL expression upregulation in bone matrix while concurrently downregulating OPG secretion, thereby accelerating osteoclast formation and enhancing their activity, which promotes bone loss. This mechanism is particularly pronounced under inflammatory and metabolic dysregulation conditions. In DOP patients, elevated GDF-15 levels are related to increased inflammatory factors, which further activate osteoclasts and exacerbate bone resorption (35, 39). Moreover, GDF-15 indirectly influences osteoclast activity by modulating immune cells, specifically by affecting the polarization state of the monocyte–macrophage system. It facilitates macrophage polarization towards the M2 phenotype while concurrently diminishing pro-inflammatory M1 macrophage activity, thereby partially modulating the inflammatory milieu associated with bone resorption. Despite these effects, the overall effect of GDF-15 is inclined to promote bone loss (39). This regulatory mechanism indicates that GDF-15 affects bone remodeling through direct interactions with osteoclasts and pathways that modulate immune responses.

## 4 Regulatory mechanisms of GDF-15 in DOP

### 4.1 GDF-15 and IR

The IR is a pathological condition characterized by diminished sensitivity to insulin and a consequent reduction in its biological effects, arising from the interconnection of genetic and environmental factors, among others. It is a pivotal mechanism in T2DM pathogenesis. Huang et al. demonstrated that when fracture incidence in patients with T2DM was considered as the dependent variable and IR levels as the independent variable, IR levels emerged as a risk factor for fracture occurrence (40). Insulin is vital for regulating bone metabolism, as studies suggest that it promotes osteoblast proliferation and stimulates collagen synthesis and ALP production. Insulin therapy can normalize bone mineral density, improve bone turnover markers, and enhance fracture healing (41). Consequently, IR, inadequate insulin secretion, or diminished insulin sensitivity may be among the primary causes of DOP.

In a study examining morbidly obese individuals and healthy subjects, the plasma concentrations of GDF-15 were significantly overexpressed in the obese cohort. These elevated levels demonstrated positive correlations with glucose, insulin, C-peptide, hemoglobin A1c, and HOMA-IR index, suggesting a connection between GDF-15 and IR markers (42). Another study identified elevated GDF-15 levels as a significant independent risk factor for T2DM development. Herein, serum GDF-15 levels in the T2DM group were markedly higher than in controls and were positively related to HOMA-IR (*p* < 0.05). Collectively, increased GDF-15 levels are closely related to IR severity and are vital for T2DM onset and progression (43). Metabolic syndrome (MS), characterized by a cluster of metabolic disorders: hypertension, hyperglycemia, and dyslipidemia, represents a considerable DM risk factor. IR is considered the pathological cornerstone of MS, with both conditions reinforcing each other through intricate molecular mechanisms (44). According to Mai et al., the serum levels of GDF-15 were significantly elevated in patients with MS compared to those in healthy controls. This finding suggests that GDF-15 may be vital for MS pathophysiology. Additionally, there was a significant positive correlation between serum GDF-15 levels and HOMA-IR in patients with MS. This correlation indicates that GDF-15 is closely related to metabolic abnormalities in MS and may contribute to IR onset and progression, thereby exacerbating metabolic disorders (45).

Building upon these studies, IR in DM patients can result in an imbalance in bone metabolism. In DM patients, elevated serum GDF-15 levels are positively related to IR indices, implying that GDF-15 may be involved in DOP by regulating IR. Nevertheless, current research is still restricted, and the underlying mechanisms require further exploration.

### 4.2 GDF-15 and high glucose toxicity

Hyperglycemic toxicity pertains to the chronic deleterious effects on cellular, tissue, and organ integrity resulting from prolonged elevated blood glucose levels. Under persistent hyperglycemic conditions, reducing sugars undergo non-enzymatic glycation reactions with proteins, lipids, and nucleic acids, generating heterogeneous compounds termed advanced glycation end products (AGEs). AGEs bind to their cognate receptor (RAGE), thereby amplifying bone resorption through dual mechanisms. First, by activating downstream nuclear factor-κB (NF-κB) signaling, which induces inflammatory responses within the bone microenvironment. Second, by increasing the binding affinity of RANK and RANKL on the surface of osteoclast precursors, thereby promoting osteoclast maturation. Collectively, these processes contribute to heightened bone resorption under diabetic conditions (46). Moreover, AGEs detrimentally affect bone formation by inducing osteoblast apoptosis. Experimental treatment of osteoblasts with the AGE precursor methylglyoxal significantly decreases ALP activity, osteoblast mineralization, and cellular viability, while concurrently downregulating the expression of RUNX2, a gene integral to osteoblast differentiation (47). Chronic hyperglycemia induces glycation of biomolecules and suppresses antioxidant enzyme activity, resulting in depleted systemic antioxidant capacity. This impairment diminishes free radical scavenging, thereby promoting oxidative stress. Oxidative stress represents an independent risk factor for DOP. In a high glucose–induced rat model of DOP, elevated reactive oxygen species (ROS) generation within osteoclasts promotes osteoclast differentiation and accelerates bone resorption.

GDF-15 exhibits a significant positive correlation with AGEs. In a cross-sectional study of 64 patients with type 1 or type 2 DM (T1DM or T2DM) and 28 healthy controls, serum AGE concentrations correlated positively with circulating GDF-15 levels (48). This association suggests that GDF-15 may participate in the co-regulation of bone metabolism via AGE-dependent signaling. Furthermore, another study reported significantly elevated GDF-15 mRNA expression in individuals with T2DM, with this alteration in expression being associated with increased ROS production and elevated inflammatory cytokine levels (49). Induction of senescence in human aortic endothelial cells via ionizing radiation increased GDF-15 levels in endothelial cells, accompanied by ROS production. This process activated the ERK pathway and led to oxidative stress-mediated senescence (50). In conclusion, GDF-15 is closely associated with hyperglycemia-induced oxidative stress in DM. This suggests its potential role as a mediator in osteoporosis driven by hyperglycemic toxicity, highlighting its significance in DOP development and progression.

### 4.3 GDF-15 and ferroptosis

Ferroptosis is an iron-dependent, non-apoptotic mode of regulated cell death defined by iron overload and the pathological accumulation of lipid hydroperoxides. Research indicates that ferroptosis induces osteocyte death *in vivo* and *in vitro*, thereby playing a critical role in the pathogenesis of DOP (51). An *in vitro* study demonstrated that AGEs impair osteoblast function by inducing ferroptosis, which promotes osteoporosis (52). Similarly, another *in vitro* study revealed that elevated glucose levels enhance the expression of activating transcription factor 3 in osteoblasts. This process inhibits the cystine/glutamate antiporter (Xc^−^ system), leading to reduced glutathione (GSH) levels, ROS accumulation, and ferroptosis induction. Consequently, this inhibits osteoblast differentiation and diminishes new bone formation (53). *In vivo* studies conducted by Lin et al. (54) demonstrated a significant reduction in the expression of two key ferroptosis-inhibiting proteins, SLC7A11 and GPX4, in bone tissue from a high-sugar, high-fat diet group, suggesting the potential occurrence of ferroptosis in the bone tissue of diabetic bone loss rats. Moreover, exposure of mouse osteoblast precursor cells (MC3T3-E1) and primary bone marrow mesenchymal stem cells to high-glucose and high-fat (HGPA) conditions, characterized by glucotoxicity and lipotoxicity, significantly reduced mRNA and protein expression levels of the osteogenic markers Runx2 and ALP, with a decrease in mineralized nodule formation. This suggests that HGPA conditions impair osteoblast function. Notably, pretreatment with the ferroptosis inhibitor Fer-1 or the iron chelator DFO prevented HGPA-provoked cell death and reversed the downregulation of SLC7A11 and GPX4 (54). Collectively, ferroptosis is involved in the pathogenesis of DM-associated osteoporosis.

The intricate relationship between GDF15 and ferroptosis has attracted considerable scholarly interest. The regulatory role of GDF15 in ferroptosis is initially evident in its effect on iron metabolism. Hepcidin, a pivotal iron-regulating hormone secreted by the pancreas, kidneys, and heart, is crucial in maintaining serum iron levels and tissue iron distribution. Research has demonstrated that GDF15 can induce iron overload by inhibiting hepcidin expression (55). Specifically, increased GDF15 concentrations are inversely correlated with hepcidin levels (56), providing essential evidence for GDF15’s influence on ferroptosis through iron metabolism. In a study on colorectal cancer, GDF15 inhibition enhanced the chemotherapeutic efficacy of 5-fluorouracil by modulating ferroptosis-related pathways, including SLC7A11/GSH/GPX4. This suggests that GDF15 may affect cancer cell survival and chemotherapy sensitivity by regulating ferroptosis (57). In spinal cord injury models, GDF-15 is vital for modulating iron-dependent cell death by stabilizing the p62-kelch-like ech-associated protein 1-nuclear factor erythroid 2-related factor 2 (Nrf2) signaling pathway, thereby alleviating neuronal damage and facilitating motor function recovery. This underscores its potential as a therapeutic target for spinal cord injury (58). In a model of neuronal injury induced by oxygen–glucose deprivation/reoxygenation, GDF-15 overexpression significantly reduced inflammatory cytokine and ferroptosis marker levels. This reduction was achieved through inhibiting endoplasmic reticulum stress-induced ferroptosis, further highlighting the neuroprotective potential of GDF-15 (59). In summary, existing research supports the involvement of ferroptosis in diabetes-related bone damage and the role of GDF-15 in regulating iron metabolism in specific non-bone tissues. However, whether GDF-15 regulates osteocyte ferroptosis in diabetic osteoporosis via a hepcidin-dependent mechanism remains speculative and requires direct experimental validation.

### 4.4 GDF-15 and inflammation

Chronic low-grade inflammation significantly contributes to DM pathogenesis and its related complications (60). Macrophages, which are essential immune cells in the human body, have been demonstrated to readily polarize towards the pro-inflammatory M1 phenotype under prolonged hyperglycemia, resulting in the substantial release of inflammatory mediators that exacerbate the progression of inflammation (61, 62). Chronic hyperglycemia induces aberrant macrophage polarization and amplifies inflammation through multiple mechanisms. First, elevated glucose levels significantly diminish the body’s antioxidant capacity, thereby enhancing ROS production in monocytes and macrophages. ROS acts as a pivotal mechanism that promotes macrophage polarization towards the M1 phenotype and triggers the release of pro-inflammatory factors (63). Second, AGEs present in the diabetic microenvironment interact with receptors on the surface of monocytes and macrophages, further stimulating these cells to secrete inflammatory mediators, including IL-1β/6, and TNF-α (64). These pro-inflammatory factors activate the pro-inflammatory transcription factor NF-κB (65). The RANKL, which is related to NF-κB signaling, is vital for osteoclastogenesis and is integral to pathological processes by influencing bone metabolism (66). In a study involving 134 elderly patients with T2DM, serum IL-1β/6 levels were significantly elevated in groups experiencing bone loss and osteoporosis compared to those with normal bone mass. These elevated inflammatory markers were also correlated with anomalies in bone metabolism indicators, including osteocalcin and 25-hydroxyvitamin D3 (67). In conclusion, DOP is regarded as an inflammation-associated variant of osteoporosis, wherein inflammatory factors regulate its onset and progression.

GDF-15 is vital to the chronic inflammatory state associated with DM. In models of diabetic nephropathy, GDF-15 demonstrates anti-inflammatory properties by inhibiting neural precursor cell-expressed developmentally downregulated gene 4-like -mediated IκB kinase (IKK)/NF-κB signaling pathway, thereby directly reducing local inflammation by preventing ubiquitin-mediated IKK degradation and the subsequent decrease in NF-κB activation (68). Nonetheless, excessive GDF-15 activity may aggravate inflammation in DM and MS. Research indicates that aberrant GDF-15 expression in these conditions is related to the suppression of iron transport and Nrf2 genes. This interaction leads to increased oxidative stress, thereby intensifying inflammatory responses and exacerbating the chronic inflammatory state of DM (49). Clinically, serum GDF-15 levels were significantly elevated in patients with MS compared to controls. Furthermore, serum GDF-15 levels are positively correlated with inflammatory markers: monocyte chemoattractant protein-1 (MCP-1) and IL-6, with IL-6 identified as an independent factor related to serum GDF-15 levels (45). Furthermore, when interpreting the clinical significance of GDF-15 in patients with diabetes, the impact of renal function cannot be overlooked. GDF-15 levels are often elevated in chronic kidney disease and diabetic nephropathy; therefore, if studies do not adequately adjust for estimated glomerular filtration rate (eGFR) or chronic kidney disease (CKD) status, it is difficult to determine whether the elevation reflects the bone disease itself or a broader renal-metabolic stress burden (69, 70). In summary, GDF-15 may participate in the progression of DOP by regulating inflammatory processes; its specific mechanisms of action and context-dependence warrant further investigation.

## 5 Summary and outlook

The pathogenesis of DOP is intricate, encompassing factors such as metabolic abnormalities, inflammatory responses, and bone cell dysfunction. GDF-15, as a multifunctional regulator, affects bone cell proliferation, differentiation, and apoptosis and indirectly disrupts the balance of bone remodeling by modulating inflammation and metabolism, thereby facilitating bone loss and highlighting its pivotal role in disease progression. Nonetheless, various studies report discrepancies in the specific mechanisms and magnitude of its effects. Some studies indicate that GDF-15 enhances osteoclast activity and accelerates bone resorption, whereas others highlight its anti-inflammatory effects and protective role for osteocytes. This apparent contradiction highlights the spatiotemporal functional specificity of GDF-15 and its multifaceted roles across different pathological stages. A comprehensive analysis of its bidirectional regulatory effects in the broader context of physiological and pathological dynamics is vital to avoid one-sided interpretations.

Currently, the relevant evidence still has several limitations. First, most clinical studies employ a cross-sectional design, making it difficult to establish a causal relationship. Second, existing studies generally have small sample sizes and high heterogeneity among study participants, which limits the comparability of results across different studies. Third, circulating GDF15 levels are influenced by various factors, including age, renal function, inflammatory status, frailty, and antidiabetic medications, complicating their interpretation as a bone-specific biomarker. Fourth, although some experimental studies suggest that GDF-15 may be involved in osteoclastogenesis, stress responses, and iron-related biological processes, evidence regarding its direct mechanisms in diabetic bone tissue remains limited. Finally, the classic GFRAL receptor is primarily expressed in the brainstem, which means that the potential mechanisms of GDF-15’s action in peripheral bone tissue remain to be further elucidated. Therefore, the current evidence is more hypothesis-generating than conclusive.

Therefore, in future research, GDF-15 should be regarded as a candidate biomarker with potential clinical value and a possible mechanistic node, rather than a validated therapeutic target. Its translational application in DOP still requires more rigorous longitudinal studies and validation of bone-specific mechanisms (71). Researchers must foster multidisciplinary collaboration by integrating insights from molecular biology, immunology, and metabolism to achieve a holistic understanding of GDF-15 function. Such collaborative efforts are expected to facilitate significant advancements in the etiology research and development of therapeutic strategies for this condition, thereby enhancing bone health and quality of life for patients.
